# Self-Determination for Nurses

**Published:** 1919-06-14

**Authors:** 


### SELF-DETERMINATION FOR NURSES.

The College of Nursing, like every great institution, is designed to fulfil the requirements of the future as well as those of to-day. England's venerable universities—Oxford and Cambridge—had their small beginnings in centuries long past, but they were founded on a principle that admitted of expansion. It is highly probable that the average nurse, like the average student, is too busily occupied with present and immediate concerns to give serious attention to developments that may take place two or ten years hence. Nevertheless it is incumbent on the responsible authorities to take a long view.

What of to-morrow? At the moment there are two Nurses' Registration Bills before Parliament. One of these is backed by fourteen thousand trained nurses whose names, addresses, and credentials are open for inspection. The other is supported by an unknown quantity. The promoters claim a membership of thirty thousand, and their refusal to supply any evidence, or any detailed information whatever, as to the identity of the members is an attitude which lends itself to suspicion. I am suspicious. Furthermore, I am perfectly satisfied in my own mind that the reason for concealment is the nakedness of the land. The Central Committee depends for success on the blare of trumpets.

Let it not be forgotten that the blare of trumpets may win. It is on record that decisive battles have been won by noise, and that armed battalions have been routed by the beating of drums. History has often a way of repeating itself. Very few Members of Parliament—extremely few—know anything, or care anything, which of the Nurses' Registration Bill becomes law. Neither of them cuts any figure in current politics, and the attitude of the majority is, "Let us get one of those Bills through, and be done with the business." Nor is this surprising. It would indeed be surprising if it were otherwise.

But the Bills before Parliament intimately concern the nursing profession for reasons which I shall endeavour to demonstrate. I have frequently stated, and I repeat, that it can make absolutely no difference to the individual whether she is able to describe herself as a trained nurse or a registered nurse so long as nondescripts of every kind are permitted to enter the field in free competition. When a public appointment is vacant the applicant must produce her credentials, and this obligation will continue. Neither Bill, in fact, has said the last word on the subject of Registration. If there is to be progress there must be amending Bills in the future. Hence there is much more than meets the eye in the present conflict. Whichever Bill passes into law will be instrumental in establishing for a very considerable time a State-appointed Nursing Council invested with wide powers, and entrusted with grave responsibilities, and the question at issue which is serious is not what benefits State Registration will confer but what powers are to be exercised by this Council. It is a case of the devil you know or the devil you don't know.

The "Times" Correspondence.

The letter written to the *Times* by Mr. Herbert J. Paterson on behalf of the Central Committee is to me an extremely interesting document. This was republished in last week's Hospital. In the first place he admits that of the 30,000 which his Bill purports to represent over 20,000 are doctors. What a revelation! Having for the first time laid bare the fact that two-thirds of his claim lie outside the profession, and failing to offer evidence that the other third consists of the genuine article, he lamely observes "The question of numbers is, however, a side issue." If numbers do not count may I inquire what counts? The only other thing that is left is the blare of the trumpets.

Mr. Paterson's letter is the only frank statement I have yet seen emanating from the Central Committee, and this is why it interests me so deeply. I have sought and searched in vain for other physical phenomena than sound. Mr. Paterson throws up the sponge as regards figures. They are a mere side issue. And then he proceeds to enunciate a doctrine which I regard as a thoroughly sound working basis. "Nursing," he says, "is a profession, and as such should have self-government by professional persons." To this I utter a loud Amen. Does Mr. Paterson suggest a Referendum? And how does he propose to dispose of the vote of the fourteen thousand trained nurses whose names, addresses, and qualifications are on view?

Trumpets and drums have their uses, but not in the domain of argument. The College of Nursing has not failed to act and to act with vigour and determination at this crucial hour to remove the grave danger of finding itself tricked. I do not suggest that a shouting party should be sent to the gallery of the House of Lords, or that nurses should scream in the streets. But I hold the view that a deputation of the College should carry to the Bar of the House of Commons a petition signed by fourteen thousand British nurses setting out the incontrovertible facts. The enemy supplies the text, "Nursing is a profession, and as such should have self-government." It is a good war-cry and will appeal to Parliament. It will appeal to the world. The College has nothing to conceal, nothing to apologise for. No Bill is better than a bad Bill, and the Central Committee's Bill, like its advocates' statements, are a tissue of misrepresentations. If British nurses are to have new masters let them be those of their own choice rather than a self-appointed oligarchy. If they are not suffered to choose their own, then let them carry on for the present with the old school—the devil they know.

IERNE.

